# An Interactive Image Segmentation Method in Hand Gesture Recognition

**DOI:** 10.3390/s17020253

**Published:** 2017-01-27

**Authors:** Disi Chen, Gongfa Li, Ying Sun, Jianyi Kong, Guozhang Jiang, Heng Tang, Zhaojie Ju, Hui Yu, Honghai Liu

**Affiliations:** 1 School of Machinery and Automation, Wuhan University of Science and Technology, Wuhan 430081, China; chendisi123@126.com (D.C.); sunying6505@hotmail.com (Y.S.); 15697188659@wo.com.cn (J.K.); whjgz@wust.edu.cn (G.J.); cds20161101@163.com (H.T.); 2 School of Computing, University of Portsmouth, Portsmouth PO1 3HE, UK; zhaojie.ju@port.ac.uk (Z.J.); hui.yu@port.ac.uk (H.Y.); honghai.liu@port.ac.uk (H.L.)

**Keywords:** image segmentation, Gibbs Energy, min-cut/max-flow algorithm, sparse representation

## Abstract

In order to improve the recognition rate of hand gestures a new interactive image segmentation method for hand gesture recognition is presented, and popular methods, e.g., Graph cut, Random walker, Interactive image segmentation using geodesic star convexity, are studied in this article. The Gaussian Mixture Model was employed for image modelling and the iteration of Expectation Maximum algorithm learns the parameters of Gaussian Mixture Model. We apply a Gibbs random field to the image segmentation and minimize the Gibbs Energy using Min-cut theorem to find the optimal segmentation. The segmentation result of our method is tested on an image dataset and compared with other methods by estimating the region accuracy and boundary accuracy. Finally five kinds of hand gestures in different backgrounds are tested on our experimental platform, and the sparse representation algorithm is used, proving that the segmentation of hand gesture images helps to improve the recognition accuracy.

## 1. Introduction

**Figure 1:**
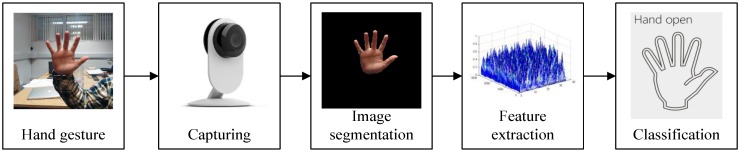
Process of hand gesture recognition.

Hand gesture recognition, utilized in visual input of controlling computers, is one of the most important aspects in human-computer interaction [1]. Compared with the traditional input methods, such as mice, keyboards and data gloves [2,3], the use of hand gestures to control computers will greatly reduce the user’s learning curve and further expand the application scenario. To achieve hand gesture control [4], many research achievements have been conducted by the pioneers in the field. Sophisticated data gloves can capture every single movement of finger joints by highly sensitive sensors [5,6] and store the hand gesture data. The hand gesture recognition process based on computer vision is illustrated in Figure 1. However, some essential problems have yet to be solved. Firstly, the vision-driven hand gesture recognition method is highly dependent on the sensibility of image sensors, therefore the relatively poor image quality hinders its development. Secondly, the image processing algorithms are not robust as they supposed to be, some of which cannot meet the demand to finish the segmentation correctly, while others fulfill the accuracy demands, but require too many human interactions [7], which are not efficient in real applications.

To address the above problems, with the cutting edge technologies, the image sensor industry has mushroomed recently. On the one hand, new kinds of image sensors, like the Microsoft Kinect 2.0, or Asus Xtion, have come into the commercial market [8], and the innovative infrared camera [9] makes it possible obtain depth information from image sensors. On the other hand, innovations in image processing algorithms have made them capable of segmenting accurate hand gestures, promoting in turn the accuracy of classifiers to ascribe gestures into different patterns.

The image segmentation is an important stage in the whole hand gesture recognition process, and several well-known segmentation methods have been proposed to meet different image segmentation demands. For example, in the graph cut method [10], proposed by Boykov and Jolly, the main idea was to divide one image into “object” and “background”. A gray scale histogram was established to describe the distribution of gray scale, and then a cut was drawn to divide the object and background. Max-flow/min cut algorithm was applied to minimize the energy function of one cut, and the segmentation was achieved by this minimized cut. These algorithms not only focus on the whole image, but also take every morphological detail into account. Random walker [11,12] is another supervised image segmentation method, where the image is viewed as an electric circuit. The edges are replaced by passive linear resistors, and the weight of each edge equals the electrical conductance. It proved to perform better segmentation compared with the graph cut method. Gulshan et al. [13] proposed an interactive image segmentation method, which regarded shape as a powerful cue for object recognition, making the problem well posed. The use of geodesic-star convexity made it have a much lower error rate compared with Euclidean star-convexity.

In the process of hand gesture recognition [13], the feature extraction is also very important. The image feature methods such as HOG [14], Hu invariant [15] and Haar [16] are used. In this paper, as for classifier and template matching algorithms, the sparse representation will be applied, since it requires much less sample for training. With the intention of recognising five different hand gestures, according to the dataset of hand gesture images, a dictionary will also be built. Then the K-SVD [17] algorithm is adapted for sample training, and the algorithm will be evaluate and compared with other methods.

## 2. Modelling of Hand Gesture Images

**Figure 2:**
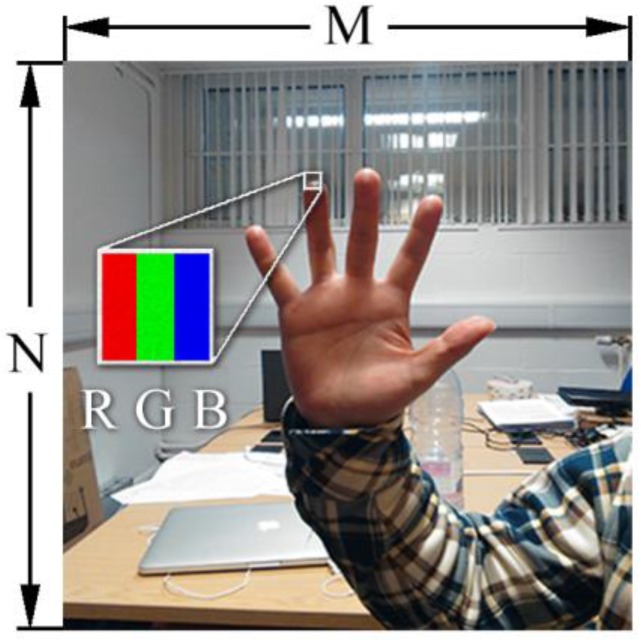
The RGB format hand gesture image.

In order to optimize the segmentation, the human visual system was carefully studied. Our eyes usually got a fuzzy picture of the whole scene at first, and then the saccadic eye movements [18] help us to obtain the details of regions of interest. With the inspiration of the human visual system, we used the Gaussian Mixture Model (GMM) [19] to get an overall view the color distributions of the image. Since the color images are mainly represented in digital formats, with tens of thousands of pixels in one image made up of red, green and blue sub-pixels, as shown in Figure 2, an M × N × 3 array was applied to store the color information in one image, where M is the horizontal resolution and N is the vertical.

### 2.1. Single Gaussian Model

The single Gaussian distribution, also known as the normal distribution [20], was proposed by the French scientist Moivre in 1733. The probability density function of a single Gaussian distribution is given by the formula:

(1) $$p(x)=\frac{1}{\sqrt{2\pi }\sigma }\exp\left(-\frac{{\left(x-\mu \right)}^{2}}{2\sigma^{2}}\right)$$

where *μ* is the mathematical expectation or the mean, *σ* is the covariance of Gaussian distribution, and exp denotes the exponential function. For convenience, the single Gaussian distribution is usually denoted as:

(2) $$X\sim N(\mu ,\sigma^{2})$$

The single Gaussian distribution formula is capable of dealing with gray scale pictures, because the variable *x* has only one dimension. One color image is an M × N × 3 array, so any element ***x****_i_* in dataset $X=\left\{x_{1},x_{2},\ldots ,x_{n}\right\}$ should be at least 3-dimensional. To address this problem, the concept of the multi-dimensional Gaussian distribution is introduced. The definition of *d* dimensional Gaussian distribution is:

(3) $$N(x;\mu ,\Sigma )=\frac{1}{\sqrt{{\left(2\pi \right)}^{d}\left|\Sigma \right|}}\exp\left[-\frac{{\left(x-\mu \right)}^{T}\Sigma^{-1}(x-\mu )}{2}\right]$$

where ***μ*** is a *d* dimensional vector, and as for the RGB model, each component of ***μ*** represents the average red, green and blue color density value. Σ is the covariance matrix and $\Sigma^{-1}$ is its inverse matrix. (***x*** − ***μ***)*^T^* is the transposed matrix of (***x*** − ***μ***). To simplify Equation (3) above, *θ* is introduced to represent the parameters ***μ*** and Σ, then the probability density function of the *d* dimensional Gaussian distribution can be written as:

(4) p(x)=N(x;θ).

According to the law of large numbers, every pixel is one sample of the real scene. When the resolution is high enough, the average color density could be estimated.

### 2.2. Gaussian Mixture Model of RGB Image

**Figure 3:**
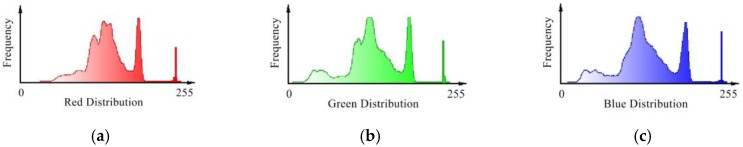
Color distributions of the gesture image. (**a**) Red distribution; (**b**) green distribution; (**c**) blue distribution.

In reality, the color distributions of the gesture image in Figure 2 can be represented by three histograms [21], shown in Figure 3. With independent red, green and blue distributions shown in Figure 3, we can notice that the gesture image cannot be exactly described by one single Gaussian model. But there are about five peaks in each histogram, so five single Gaussian models should be applied in gesture image modelling.

GMM is introduced to approximate the continuous probability distribution by increasing the number of single Gaussian models. The probability density function of GMM with k mixed Gaussian models becomes:

(5) $$p(x)=\sum_{i=1}^{k}\pi_{i}p_{i}(x;\theta_{i})$$

(6) $$p(x)=\sum_{i=1}^{k}\pi_{i}N_{i}(x;\mu_{i},\Sigma_{i}),$$

where i∈{1,2,…,k} shows which single Gaussian model the component belongs to. $\pi_{i}$ is the mixing coefficients of *k* mixed component [22] or the prior probability of ***x*** belonging to the *i*-th single Gaussian model, and $\sum_{i=1}^{k}\pi_{i}=1$. $p_{i}(x;\theta_{i})$ is the probability density function of the *i*-th single Gaussian model, parameterized by $\mu_{i}$ and $\Sigma_{i}$ in $N_{i}(x;\mu_{i},\Sigma_{i})$. Θ is introduced as a parameters [23] set, {$\pi_{1},\pi_{2},\ldots ,\pi_{k},\theta_{1},\theta_{2},\ldots ,\theta_{k}$}, to denote $\alpha_{i}$ and $\theta_{i}$. 

As mentioned above, one RGB hand gesture image could be described in the dataset $X=\left\{x_{1},x_{2},\ldots ,x_{n}\right\}$, and if we regard ***X*** as a sample, its probability density is:

(7) $$p(X;\Theta )=\prod_{j=1}^{n}p(x_{j};\Theta )=L(\Theta ;X),x_{j}\in X,$$

where L(X;Θ) is called likelihood function of parameters given the sample ***X***. Then we hope to find a set of parameter Θ to finish modelling. According to maximum likelihood method [24], our next task is to find $\hat{\Theta }$ where:

(8) $$\hat{\Theta }=\underset{\Theta }{\arg\max}\;L(\Theta ;X).$$

The function L(Θ;X) and L(X;Θ) have the same equation form, but considering now we are going to use ***X*** to estimate Θ, the Θ becomes variables and ***X*** are the fixed parameters, it is denoted in the second form. The value of p(X;Θ) is usually too small to be calculated by computer, so we are going to replace it with the log-likelihood function [25]:

(9) $$\ln(L(\Theta ;X))=\ln\left[\prod_{j=1}^{n}p(x_{j};\Theta )\right]$$

(10) $$=\sum_{j=1}^{n}\ln\left[\sum_{i=1}^{k}\pi_{i}p_{i}(x_{j};\theta_{i})\right].$$

### 2.3. Expectation Maximum Algorithm

After establishing the Gaussian mixture model of a RGB hand gesture image, there are still several parameters that need to be estimated. The expectation maximum (EM) algorithm [26] is introduced for the subsequent calculations. The EM algorithm is a method of acquiring the parameters set Θ in the maximum likelihood method. There are two steps in this algorithm, called the E-step and M-step, respectively. To start the E-step we will introduce another probability *Q_i_*(***x****_j_*). It is a posterior probability of *π_i_*, in another words, the posterior probability of each ***x****_j_* belonging to the *i*-th single Gaussian model, from the dataset ***X***.

(11) $$Q_{i}(x_{j})=\frac{\pi_{i}p_{i}(x_{j};\theta_{i})}{\sum_{t=1}^{k}\pi_{t}p_{t}(x_{j};\theta_{t})},$$

where the definition of $Q_{i}(x_{j})$ is given according to Bayes’ theorem, and $\sum_{i=1}^{k}Q_{i}(x_{j})=1$. Then we use Equation (11) to modify the log-likelihood function in (10):

(12) $$\ln(L(\Theta ;X))=\sum_{j=1}^{n}\ln\left[\sum_{i=1}^{k}Q_{i}(x_{j})\frac{\pi_{i}p_{i}(x_{j};\theta_{i})}{Q_{i}(x_{j})}\right]$$

(13) $$\geq \sum_{j=1}^{n}\sum_{i=1}^{k}Q_{i}(x_{j})\ln\left[\frac{\pi_{i}p_{i}(x_{j};\theta_{i})}{Q_{i}(x_{j})}\right].$$

From (12) to (13), the Jensen’s inequality have been applied, since $ln^{\prime \prime }(x)=-\frac{1}{x^{2}}\leq 0$, it is concave on its domain. Then:

(14) $$\ln\left[\sum_{i=1}^{k}Q_{i}(x_{j})\frac{\pi_{i}p_{i}(x_{j};\theta_{i})}{Q_{i}(x_{j})}\right]\geq \sum_{i=1}^{k}Q_{i}(x_{j})\ln\left[\frac{\pi_{i}p_{i}(x_{j};\theta_{i})}{Q_{i}(x_{j})}\right],$$

Maximizing Equation (13) guarantees that ln(L(Θ;X)) is maximized. The iteration of an EM algorithm estimating the new parameters in terms of the old parameters is given as follows:

- *Initialization*: Initialize $\mu_{i0}$ with random numbers [27], and the unit matrices are used as covariance matrices $\Sigma_{i0}$ to start the first iteration. The mixed coefficients or prior probability is assumed as $\pi_{i0}=\frac{1}{k}$.
- *E-step*: Compute the posterior probability of $\pi_{i}$ using current parameters:
  (15) $$Q_{i}(x_{j}):=\frac{\pi_{i}p_{i}(x_{j};\theta_{i})}{\sum_{t=1}^{k}\pi_{t}p_{t}(x_{j};\theta_{t})}=\frac{\pi_{i}N(x_{j};\mu_{i},\Sigma_{i})}{\sum_{t=1}^{k}\pi_{t}N(x_{j};\mu_{t},\Sigma_{t})}$$
- *M-step*: Renew the parameters:
  (16) $$\pi_{i}:=\frac{1}{n}\sum_{j=1}^{n}Q_{i}(x_{j})$$
  (17) $$\mu_{i}:=\frac{\sum_{j=1}^{n}Q_{i}(x_{j})x_{t}}{\sum_{j=1}^{n}Q_{i}(x_{j})}$$
  (18) $$\Sigma_{i}:=\frac{\sum_{j=1}^{n}Q_{i}(x_{j})(x_{j}-\mu_{i}){\left(x_{j}-\mu_{i}\right)}^{T}}{\sum_{j=1}^{n}Q_{i}(x_{j})}$$

For most hand gesture images, the number of iterations is usually defined as a certain number. In order to improve the segmentation quality and to take account of the efficiency, the number of iterations should be 8 [28].

## 3. Interactive Image Segmentation

**Figure 4:**
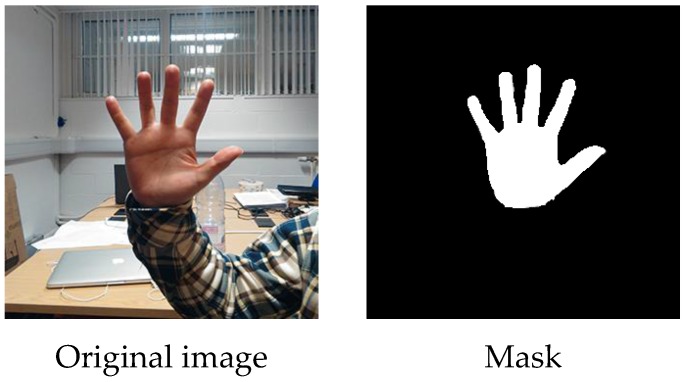
The mask.

The modelling method discussed previously provides a universal way of dealing with hand gesture images. To segment the digital images, a mask is introduced as shown in Figure 4, which is a binary bitmap denoted as ***α***. By introducing it, we changed the segmentation problem into a pixels labelling problem. As *α_j_* ∈ {1,0}, the value 0 is taken for labelling background pixels and 1 for foreground pixels.

To deal with the GMM tractably, we introduce two independent *k*-component GMMs, one for the foreground modelling and one for the background modelling. Each pixel ***x****_j_*, either from the background or the foreground model, is marked as *α_j_* = 1 or 0. The parameters of each component become: *θ_i_* = {*π_i_*(*α_j_*), *μ_i_*(*α_j_*), *Σ_i_*(*α_j_*); *α_j_* = 0,1, *i* = 1, …, *k*}.

### 3.1. Gibbs Random Field

The overall color modelling completes the first step in our human visual system, to take every detail of the image into account, Gibbs random field (GRF) [29] is introduced. GRF is defined as:

(19) $$P(A=a)=\frac{1}{Z(T)}\exp(-\frac{1}{T}E(\alpha )),$$

Here, P(A=a) gives the probability of the system ***A*** being in the state ***a***. *T* is a constant parameter, whose unit is temperature in physics, and usually its value is 1. Z(T) is the partition function, and:

(20) $$Z(T)=\sum_{a\in A}\exp(-\frac{1}{T}E(a)),$$

where, E(α) is interpreted as the energy function of the state ***a***, to apply GRF in image segmentation, the Gibbs Energy [30] can be defined as follows:

(21) E(α)=E(α,Θ,X)=E(α,i,θ,X)=U(α,i,θ,X)+V(α,X)

The term U(α,i,θ,X), also called regional term, is defined taking account of GMM. It indicates the penalty of $x_{j}$ being classified in the background or foreground:

(22) $$U(\alpha ,i,\theta ,X)=\sum_{j=1}^{n}-\ln[p_{i}(x_{j})\times \pi_{i}(\alpha_{j})],$$

(23) $$=\sum_{j=1}^{n}\left\{-\ln[\pi_{i}(\alpha_{j})]-\ln[\frac{1}{2}\ln\left|\Sigma_{i}(\alpha_{j})\right|]+\frac{1}{2}{\left[x_{j}-\mu_{i}(\alpha_{j})\right]}^{T}\Sigma_{i}{\left(\alpha_{j}\right)}^{-1}[x_{j}-\mu_{i}(\alpha_{j})]\right\}.$$

and V(α,X), which is the boundary term, which is defined to describe the smoothness between pixel $x_{u}$ and its neighbour pixels $x_{v}$ in the pixel set ***N***:

(24) $$V(\alpha ,X)=\gamma \sum_{x_{u},x_{v}\in N}\left[\alpha_{u}\neq \alpha_{v}\right]\exp(-\beta {\left\|x_{u}-x_{v}\right\|}^{2}),$$

where the constant γ was obtained as 50 by optimizing the efficiency over training. $\left[\alpha_{u}\neq \alpha_{v}\right]$ is an indicator function taking values 0 or 1, by judging the formula inside. *β* is a constant, which represents the contrast of the pixel set ***N***, to adjust the exponential term. E(x) in the equation below is the expectation:

(25) $$\beta =\frac{1}{2\underset{x_{u},x_{v}\in N}{E}[{\left(x_{u}-x_{v}\right)}^{T}(x_{u}-x_{v})]}$$

### 3.2. Automatical Seed Selection

**Figure 5:**
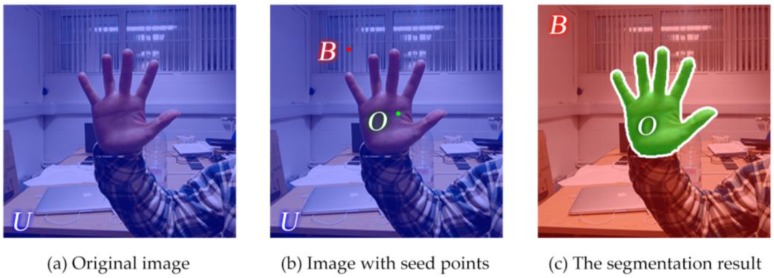
The relationships between three pixel sets.

Until now all the constants have been defined. To begin with, all the pixels in the picture are automatically marked as undefined and labeled ***U*** [31]. ***B*** is the background seed pixel set and ***O*** is the foreground seed set. After the training over training set ***X***, the set ***O*** is obtained as the segmentation result and O⊂U. Three pixel sets are shown in Figure 5.

**Figure 6:**
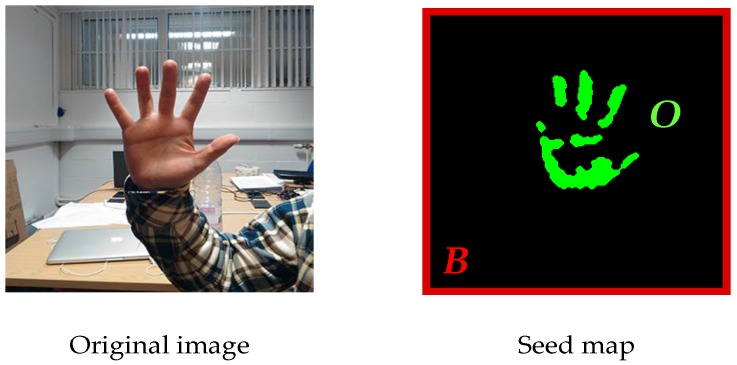
The result of automatic seed selection.

To achieve the segmentation automatically, we propose an initial seeds selection method in hand gesture images. Considering that the human skin color has an elliptical distribution in *YCbCr* color space [32], the image is transformed from RGB color space to *YCbCr*, using the equation below:

(26) $$\left[\begin{array}{c} Y\\ Cb\\ Cr \end{array}\right]=\left[\begin{array}{c} 16\\ 128\\ 128 \end{array}\right]+\frac{1}{256}\cdot \left[\begin{array}{ccc} 65.738 & 129.057 & 25.06\\ -37.945 & -74.494 & 112.43\\ 112.439 & -94.154 & -18.28 \end{array}\right]\cdot \left[\begin{array}{c} r\\ g\\ b \end{array}\right],$$

where, *Y* indicates the luminance. By setting Y∈(0,80), the interference of highlights would be overcome. Then the *Cb*, *Cr* values of human skin color are located by the elliptical equations given below:

(27) $$\left\{ \begin{array}{l} \frac{{\left(x-1.6\right)}^{2}}{26.39^{2}}+\frac{{\left(y-2.41\right)}^{2}}{14.03^{2}}<1\\ \left[\begin{array}{c} x\\ y \end{array}\right]=\left[\begin{array}{cc} \cos(2.53) & \sin(2.53)\\ -\sin(2.53) & \cos(2.53) \end{array}\right]\cdot \left[\begin{array}{c} Cb-109.38\\ Cr-152.02 \end{array}\right] \end{array}\right.,$$

where, *x* and *y* are the intermediate variables. All the pixels satisfying the equations above will be marked as the foreground seeds, which belong to set ***O***. We also define the pixels on the image edges as background seeds, which belong to set *B*, because the gestures are usually located far away from the edges of the images. The result of seeds selection are displayed in Figure 6 below.

### 3.3. Min-Cut/Max-Flow Algorithm

According to the Gibbs random field, the image segmentation or pixel labelling problem equals minimizing the Gibbs energy function:

(28) $$\underset{\left\{\alpha_{j};i\in U\right\}}{\min}[\underset{i}{\min}\;E(\alpha ,i,\theta ,X)]$$

**Figure 7:**
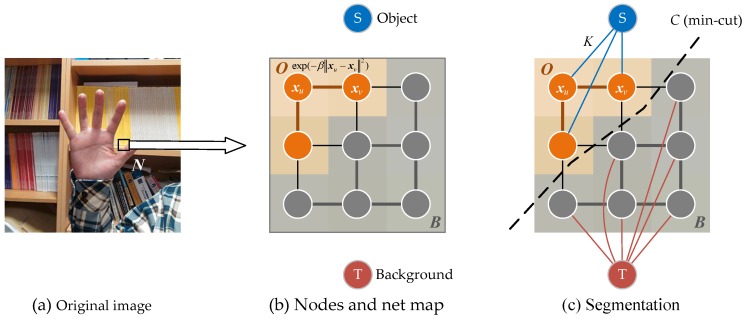
Nodes and net model.

The min-cut/max-flow algorithm [33] is proposed to finish the segmentation more accurately. The idea of this algorithm is to regard one image as a net with nodes, and each node take the place of a corresponding pixel. Apart from that, two extra nodes, *S* and *T*, are introduced, which represent “source” and “sink”, respectively. Node *S* is linked to pixels belonging to *O*, while *T* linked pixels in *B* as shown in Figure 7.

There are three kinds of links in the neighbourhood ***N***, from pixel to pixel, from pixel to *S* and from pixel to *T*, denoted as $\bar{x_{u}x_{v}},\;\bar{x_{u}S},\;\bar{x_{u}T}$. Each link is assumed with a certain weight or a cost [34] while it being cut down, which detailed in Table 1.

According to the max-flow/min-cut theorem, an optimal segmentation is defined by the minimum cut *C* as seen in Figure 7c. *C* is known as a set of $\bar{x_{u}x_{v}}$ links, so that:

(29) $$\left|C\right|=\sum_{x\in U}U(C,i,\theta ,x)+\sum_{x\in N}V(C,x)$$

(30) $$=E(C,i,\theta ,X)-\left[\sum_{x\in O}U(\alpha =1,i,\theta ,x)+\sum_{x\in B}U(\alpha =0,i,\theta ,x)\right]$$

Then the Gibbs energy could be minimized by using the min-cut defined above. The whole process of this segmentation is as follows: firstly, assign the GMM components *i* to each $x_{j}\in U$ according to the human select of the ***U*** region. Secondly, the parameters set Θ is learned from the whole pixel set ***X***. Thirdly, use the min-cut to minimize the Gibbs energy of the whole image. Then jump to the first step to start another round, and after eight times, the optimal segmentation will be achieved.

## 4. Experimental Comparison

**Figure 8:**
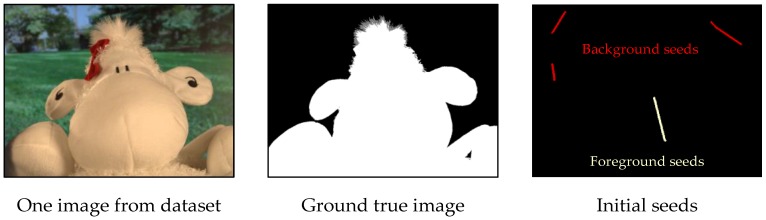
The evaluation samples from dataset.

To evaluate interactive segmentation quantitatively, an image dataset proposed by Gulshan [13], which contains 49 images from GrabCut dataset [35], 99 images from PASCAL VOC’09 segmentation challenge [36] and 3 images from the alpha-matting dataset [37] is chosen. Those images cover all kinds of shapes, textures and backgrounds. The corresponding ground true images together with the initial seeds were also included in this dataset. The initial seed maps were made up of 4 manually generated brush-strokes all in 8 pixels wide, and one for foreground and 3 for background as shown in Figure 8.

**Figure 9:**
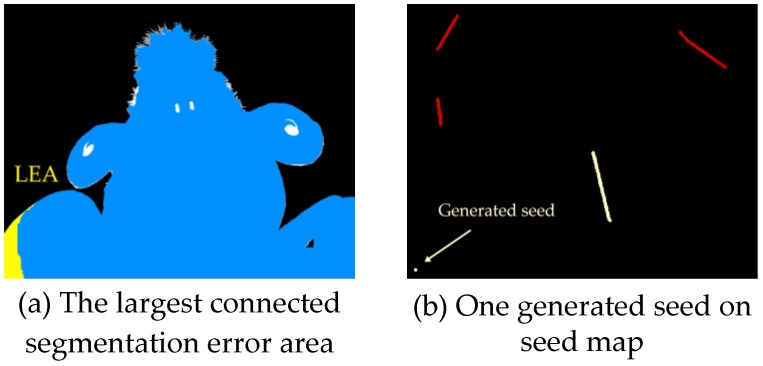
Evaluation on the dataset.

To simulate the human interactions, after the first segmentation with initial seed map, one more seed would be generated in the largest connected segmentation error area (LEA) automatically. As shown in Figure 9a, the blue area is the segmentation result of the algorithm, while the white one is the ground true segmentation and the LEA is marked in yellow. From Figure 9b, the seed is a round dot (8 pixels in diameter), generated according to the LEA. Then we update the segmentation with all the seeds. After that, this step is repeated 20 times, and a sequence of segmentations will be obtained. 

To evaluate the quality of segmentation results, we used two different methods in evaluating the region accuracy (*RA*) and boundary accuracy (*BA*). Each evaluation will be conducted to a single segmentation, and all the images in Gushan’s dataset will be tested to verify that our proposed method is suitable for interactive image segmentation.

### 4.1. Region Accuracy

The *RA* of segmentation results is evaluated by a weighted *F_β_* − *measure* [38]. Compared with normal *F_β_* − *measure*, the two terms *Precision* and *Recall* become:

(31) $$Precision^{w}=\frac{TP^{w}}{TP^{w}+FP^{w}}$$

(32) $$Recall^{w}=\frac{TP^{w}}{TP^{w}+NP^{w}}$$

where, *TP* denotes the overlap of ground true and segmented foreground pixels. *FP* is the wrongly segmented pixels compared with ground true images and *NP* represent the wrongly segmented background pixels. 

The $F_{\beta }^{w}-measure$ is defined as follows:

(33) $$RA=F_{\beta }^{w}=(1+\beta^{2})\frac{Precision^{w}\cdot Recall^{w}}{\beta^{2}\cdot Precision^{w}+Recall^{w}}$$

where, *β* signifies the effectiveness of detection with respect to a user who attaches *β* times as much importance to *Recall^w^* as to *Precision^w^*, normally *β* = 1. Then, we apply $F_{1}^{w}-measure$ to calculate the *RA* of different segmentation results. The higher *RA* is, the better the segmentation achieved is.

### 4.2. Boundary Accuracy

**Figure 10:**
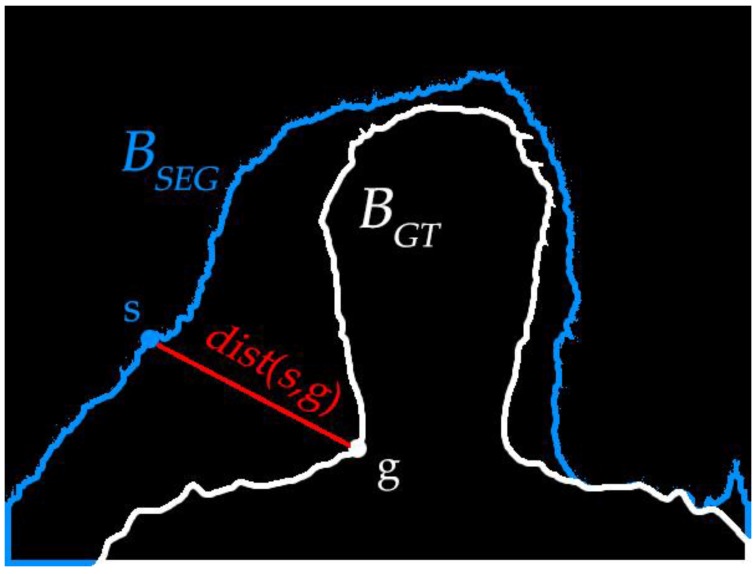
Boundary extraction.

The *BA* [39] is defined according to the Hausdorff distance. The boundary pixels of ground true image and segmented image are defined as *B_GT_* and *B_SEG_* as shown in Figure 10. 

The formula is as follows:

(34) $$BA=\frac{N(B_{SEG})+N(B_{GT})}{\sum_{s}\underset{g}{\min}(dist(s,g))+\sum_{g}\underset{s}{\min}(dist(g,s))},$$

where, *g* ∈ *B_GT_* and *s* ∈ *B_SEG_*, *dist*(**·**) denotes the Euclidean distance, *N*(**·**) is the pixel number in the set. The value of *BA* shows the segmentation accuracy of boundaries.

### 4.3. Results Analysis

**Figure 11:**
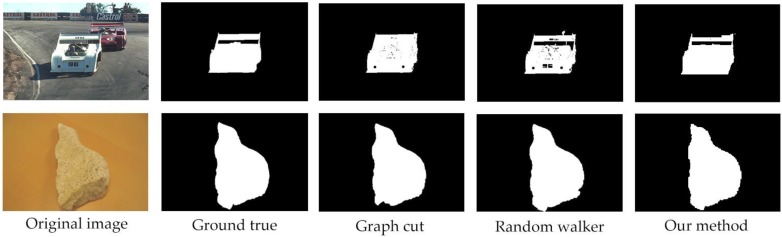
The evaluation on different algorithms.

**Figure 12:**
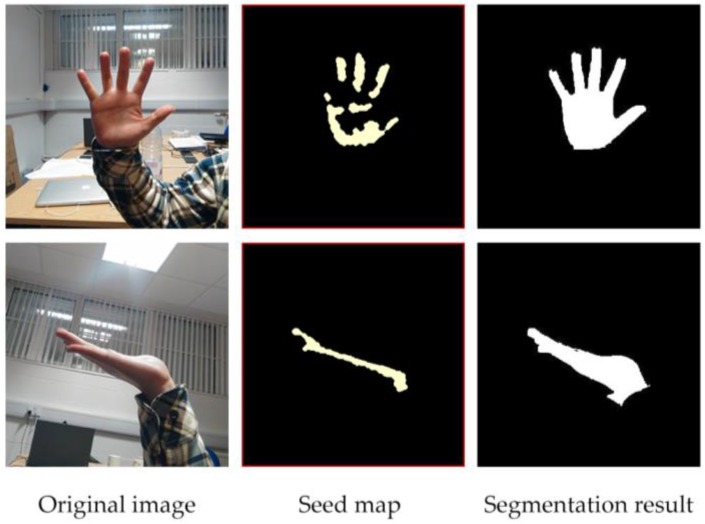
Segmentation results of our method on hand images.

We segmented the images from the dataset by graph cut and random walker as shown in Figure 11. The segmentation test of our method has been made on Gulshan’s dataset as well as our hand gesture images, and some of the results using our method on hand gesture image segmentation are shown here in Figure 12.

**Figure 13:**
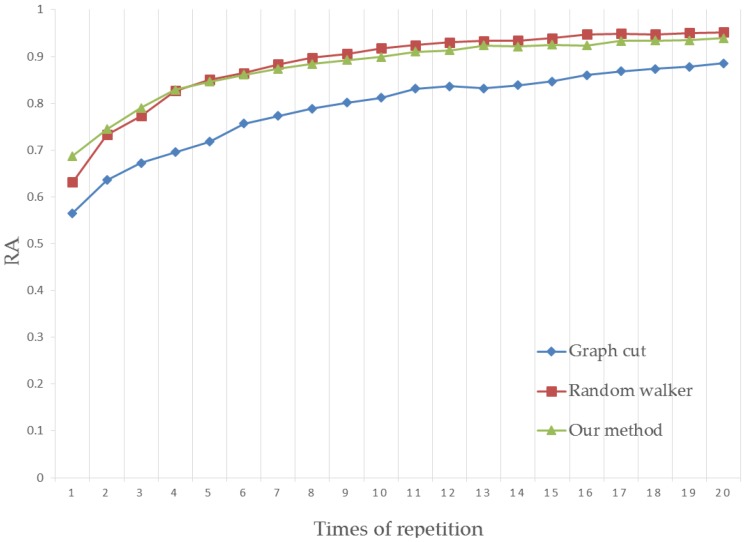
Region accuracy comparison.

**Figure 14:**
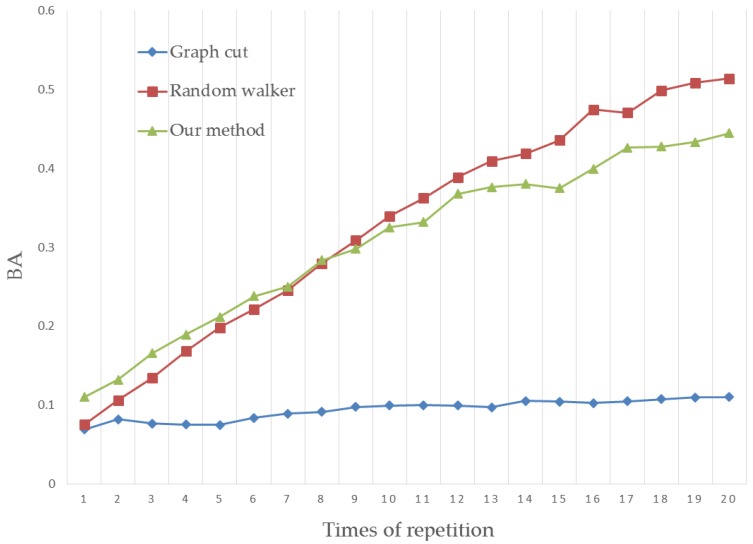
Boundary accuracy comparison.

For a more rigorous test, we tested 151 images from Gulshan’s dataset and used the human interaction simulator to perform the interactions, which generated the seeds 20 times to further refine the segmentation results. The result of each simulation step has been tested on the experiment platform. The RA and BA scores are the mean values of 151 segmentations, shown in Figure 13 and Figure 14.

From the figures above, the segmentation quality shows an increase with simulated human interactions. When the seed number becomes high, a satisfactory segmentation will be achieved. Our method obtains the best segmentation quality with few human interactions. Since the seeds are generated once automatically in human hand image segmentation, our method is suitable for human image segmentation.

## 5. Hand Gesture Recognition

**Figure 15:**
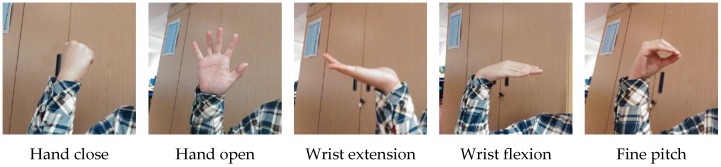
Five hand gestures for recognition.

We defined five hand gestures: hand closed (HC), hand open (HO), wrist extension (WE), wrist flexion (WF), and fine pitch (FP), as shown in Figure 15.

**Figure 16:**
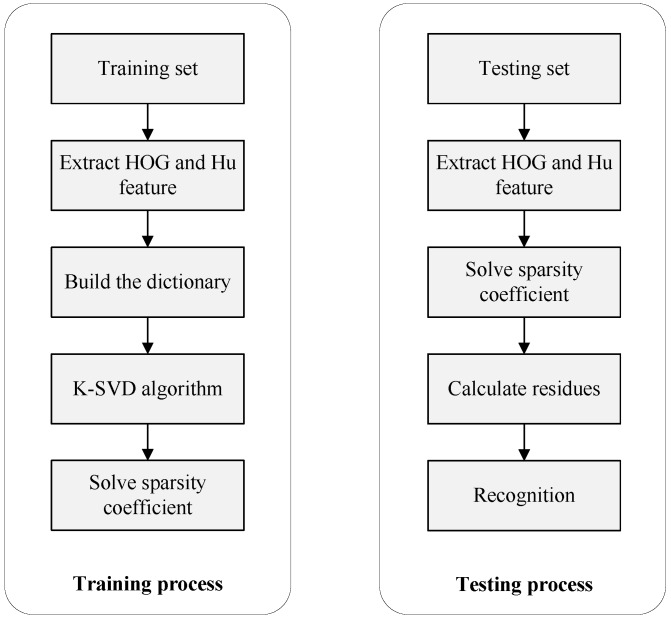
Hand gesture recognition framework.

One hundred images of each hand gesture were captured and segmented by the proposed method. We used the recognition framework in Figure 16. Each gesture takes 50 images for training and 50 for testing. To achieve a better classification, we extract HOG along with Hu invariant moments at the same weights. The K-SVD dictionary training method [40] is used to choose atoms representing [41] all features and reduce the computation costs.

We tested the recognition rates on both unsegmented hand images and segmented hand images. The recognition rates on unsegmented hand images are shown in Table 2, and the recognition rates on segmented hand images are shown in Table 3.

By segmenting the images before feature extraction, the recognition rates on those five hand gestures are increased compared with unsegmented images, according to the results in the tables above. 

## 6. Conclusions and Future Work

In conclusion, the interactive hand gesture image segmentation method can perfectly meet the segmentation demands of hand gesture images with no human interactions. The mechanism behind this method is carefully explored and deduced with the assistance of modern mathematical theories. Comparing the segmentation results of hand gestures with other popular image segmentation methods, our method can obtain a better segmentation accuracy and a higher quality, when there are limited seeds. Automatic seeds selection also helps to reduce human interactions. The segmentation work in turn improves the recognition rate. In future work, we could adapt this method to higher resolution pictures, which requires simplifying the calculation process. In seed selection, the automatic selection method could be improved to overcome various interferes, such as highlights, shadows and image distortion. Other future work will focus on improving the recognition rate by integrating the segmentation algorithm with more advanced recognition methods. 

## Figures and Tables

**Table 1:** The weight of each link.

Link Type	Weight	Precondition
$\bar{x_{u}x_{v}}$	$\exp\left(-\beta \|x_{u}-x_{v}{\|}^{2}\right)$	$x_{u},x_{v}\in N$
$\bar{x_{u}S}$	U(α=0,i,θ,X)	$x_{u}\in U$
	*K*	$x_{u}\in O$
	0	$x_{u}\in B$
$\bar{x_{u}T}$	U(α=1,i,θ,X)	$x_{u}\in U$
	0	$x_{u}\in O$
	*K*	$x_{u}\in B$
where $K=1+\underset{x_{u}\in X}{\max}\sum_{x_{u},x_{v}\in N}\exp(-\beta {\left\|x_{u}-x_{v}\right\|}^{2})$		

**Table 2:** Recognition rates on unsegmented hand images.

Gestures	Recognition Rates
Hand close	86.7%
Hand open	73.3%
Wrist extension	100%
Wrist flexion	100%
Fine pitch	66.7%
**Over all rate**	**85.3%**

**Table 3:** Recognition rates on segmented hand images.

Gestures	Recognition Rates
Hand close	93.3%
Hand open	100%
Wrist extension	100%
Wrist flexion	100%
Fine pitch	100%
**Over all rate**	**98.7%**

## References

[1] Nardi B.A. (1996). Context and Consciousness: Activity Theory and Human-Computer Interaction.

[2] Chen D.C., Li G.F., Jiang G.Z., Fang Y.F., Ju Z.J., Liu H.H. (2015). Intelligent Computational Control of Multi-Fingered Dexterous Robotic Hand. J. Comput. Theor. Nanosci..

[3] Ju Z.J., Zhu X.Y., Liu H.H. (2011). Empirical Copula-Based Templates to Recognize Surface EMG Signals of Hand Motions. Int. J. Humanoid Robot..

[4] Miao W., Li G.F., Jiang G.Z., Fang Y., Ju Z.J., Liu H.H. (2015). Optimal grasp planning of multi-fingered robotic hands: A review. Appl. Comput. Math..

[5] Farina D., Jiang N., Rehbaum H., Holobar A., Graimann B., Dietl H., Aszmann O.C. (2014). The extraction of neural information from the surface EMG for the control of upper-limb prostheses: Emerging avenues and challenges. IEEE Trans. Neural Syst. Rehabil. Eng..

[6] Ju Z., Liu H. (2014). Human Hand Motion Analysis with Multisensory Information. IEEE/ASME Trans. Mechatron..

[7] Panagiotakis C., Papadakis H., Grinias E., Komodakis N., Fragopoulou P., Tziritas G. (2013). Interactive Image Segmentation Based on Synthetic Graph Coordinates. Pattern Recognit..

[8] Yang D.F., Wang S.C., Liu H.P., Liu Z.J., Sun F.C. (2012). Scene modeling and autonomous navigation for robots based on kinect system. Robot.

[9] Wang C., Liu Z., Chan S.C. (2015). Superpixel-Based Hand Gesture Recognition with Kinect Depth Camera. Trans. Multimed..

[10] Sinop A.K., Grady L. A Seeded Image Segmentation Framework Unifying Graph Cuts and Random Walker Which Yields a New Algorithm. Proceedings of the IEEE 11th International Conference on Computer Vision (ICCV).

[11] Grady L. Multilabel random walker image segmentation using prior models. Proceedings of the IEEE Computer Society Conference on Computer Vision and Pattern Recognition (CVPR’05).

[12] Couprie C., Grady L., Najman L., Talbot H. Power watersheds: A new image segmentation framework extending graph cuts, random walker and optimal spanning forest. Proceedings of the IEEE 12th International Conference on Computer Vision (ICCV).

[13] Varun G., Carsten R., Antonio C., Andrew B., Andrew Z. Geodesic star convexity for interactive image segmentation. Proceedings of the IEEE CVPR.

[14] Ju Z., Liu H. (2011). A Unified Fuzzy Framework for Human Hand Motion Recognition. IEEE Trans. Fuzzy Syst..

[15] Xu Y., Yu G., Wang Y., Wu X., Ma Y. (2016). A Hybrid Vehicle Detection Method Based on Viola-Jones and HOG + SVM from UAV Images. Sensors.

[16] Fernando M., Wijjayanayake J. (2015). Novel Approach to Use Hu Moments with Image Processing Techniques for Real Time Sign Language Communication. Int. J. Image Process..

[17] Chen Q., Georganas N.D., Petriu E.M. Real-time vision-based hand gesture recognition using haar-like features. Proceedings of the EEE Instrumentation & Measurement Technology Conference IMTC.

[18] Sun R., Wang J.J. (2014). A Vehicle Recognition Method Based on Kernel K-SVD and Sparse Representation. Pattern Recognit. Artif. Intell..

[19] Jiang Y.V., Won B.-Y., Swallow K.M. (2014). First saccadic eye movement reveals persistent attentional guidance by implicit learning. J. Exp. Psychol. Hum. Percept. Perform..

[20] Ju Z., Liu H., Zhu X., Xiong Y. (2009). Dynamic Grasp Recognition Using Time Clustering, Gaussian Mixture Models and Hidden Markov Models. Adv. Robot..

[21] Bian X., Zhang X., Liu R., Ma L., Fu X. (2014). Adaptive classification of hyperspectral images using local consistency. J. Electron. Imaging.

[22] Song H., Wang Y. (2016). A spectral-spatial classification of hyperspectral images based on the algebraic multigrid method and hierarchical segmentation algorithm. Remote Sens..

[23] Hatwar S., Anil W. (2015). GMM based Image Segmentation and Analysis of Image Restoration Tecniques. Int. J. Comput. Appl..

[24] Couprie C., Najman L., Talbot H. (2011). Seeded segmentation methods for medical image analysis. Medical Image Processing.

[25] Bańbura M., Modugno M. (2014). Maximum likelihood estimation of factor models on datasets with arbitrary pattern of missing data. J. Appl. Econ..

[26] Simonetto A., Leus G. (2013). Distributed Maximum Likelihood Sensor Network Localization. IEEE Trans. Signal Process..

[27] Ju Z., Liu H. (2012). Fuzzy Gaussian Mixture Models. Pattern Recognit..

[28] Zhang Y., Brady M., Smith S. (2001). Segmentation of brain MR images through a hidden Markov random field model and the expectation-maximization algorithm. IEEE Trans. Med. Imaging.

[29] Song W., Cho K., Um K., Won C.S., Sim S. (2012). Intuitive terrain reconstruction using height observation-based ground segmentation and 3D object boundary estimation. Sensors.

[30] Wei S., Kyungeun C., Kyhyun U., Chee S., Sungdae S. (2012). Complete Scene Recovery and Terrain Classification in Textured Terrain Meshes. Sensors.

[31] Liao L., Lin T., Li B., Zhang W. (2008). MR brain image segmentation based on modified fuzzy C-means clustering using fuzzy GIbbs random field. J. Biomed. Eng..

[32] Kakumanu P., Makrogiannis S., Bourbakis N. (2007). A survey of skin-color modeling and detection methods. Pattern Recognit..

[33] Lee G., Lee S., Kim G., Park J., Park Y. (2016). A Modified GrabCut Using a Clustering Technique to Reduce Image Noise. Symmetry.

[34] Ning J., Zhang L., Zhang D., Wu C. (2010). Interactive image segmentation by maximal similarity based region merging. Pattern Recognit..

[35] Grabcut Image Dataset. http://research.microsoft.com/enus/um/cambridge/projects/visionimagevideoediting/segmentation/grabcut.htm.

[36] Everingham M., Van G.L., Williams C.K., Winn I.J., Zisserman A. The PASCAL Visual Object Classes Challenge 2009 (VOC2009) Results. http://host.robots.ox.ac.uk/pascal/VOC/voc2009/.

[37] Rhemann C., Rother C., Wang J., Gelautz M., Kohli P., Rott P. A perceptually motivated online benchmark for image matting. Proceedings of the CVPR.

[38] Margolin R., Zelnik-Manor L., Tal A. How to Evaluate Foreground Maps?. Proceedings of the IEEE Conference on Computer Vision and Pattern Recognition.

[39] Zhao Y., Nie X., Duan Y. A benchmark for interactive image segmentation algorithms. Proceedings of the IEEE Person-Oriented Vision.

[40] Zhou Y., Liu K., Carrillo R.E., Barner K.E., Kiamilev F. (2013). Kernel-based sparse representation for gesture recognition. Pattern Recognit..

[41] Yu F., Zhou F. (2016). Classification of machinery vibration signals based on group sparse representation. J. Vibroeng..

